# Caecal Volvulus, Untwisting the Twisted: A Case Report and Literature Review

**DOI:** 10.7759/cureus.30961

**Published:** 2022-11-01

**Authors:** Farah F Kazi, Ahmed Kazi, Arvin Kumar, Haresh Perthiani

**Affiliations:** 1 General Surgery, St. James's Hospital, Dublin, IRL; 2 Surgery, Our Lady of Lourdes Hospital, Drogheda, IRL; 3 General Surgery, Our Lady of Lourdes Hospital, Drogheda, IRL

**Keywords:** laparoscopic right hemicolectomy, caecal volvulus, emergency, peritoneum, caecopexy, volvulus, caecal, abdominal

## Abstract

Caecal volvulus is an uncommon surgical condition affecting mostly females in their second and third decade of life. It is of vital importance that the general surgeon recognises, resuscitates, diagnoses, and effectively treats these cases in a timely manner to maximise the chance of a positive outcome for the patient. Whilst there are several types of caecal volvulus, the treatment involves, in most cases, surgical intervention. There is a wide variety of surgical interventions that can be performed, ranging from caecopexy or fixation to lateral wall to performing a right hemicolectomy with primary ileocolic anastomosis. There are several factors that influence this decision and can also be based on an individual surgeon's expertise and experience. We present a case of a 21-year-old female who presented to our Emergency Department with lower abdominal pain, nausea, and vomiting. She was diagnosed with caecal volvulus with the aid of CT imaging, following which she underwent laparotomy in which caecal volvulus was noted. She underwent appendicectomy and caecopexy and was discharged after an uneventful recovery on post-operative day five and remains well on follow-up.

## Introduction

Caecal volvulus accounts for 25-40% of all cases of colonic volvulus [1]. It is defined as the axial twisting of the caecum, usually occurring in individuals with a highly mobile caecum [1]. As it is an uncommon condition, early diagnosis and prompt timely management of caecal volvulus are key in reducing associated complications. Diagnosis can be challenging due to the variability in clinical presentation, varying age groups of presentation, and potential misinterpretation of radiological imaging. While there is a wide spectrum of clinical presentations, most commonly it presents as an acute bowel obstruction secondary to rapid strangulation. As such, it is of vital importance for the general surgeon to recognise this condition in coordination with an experienced radiologist to ensure positive outcomes for the patient.

## Case presentation

**Figure 1:**
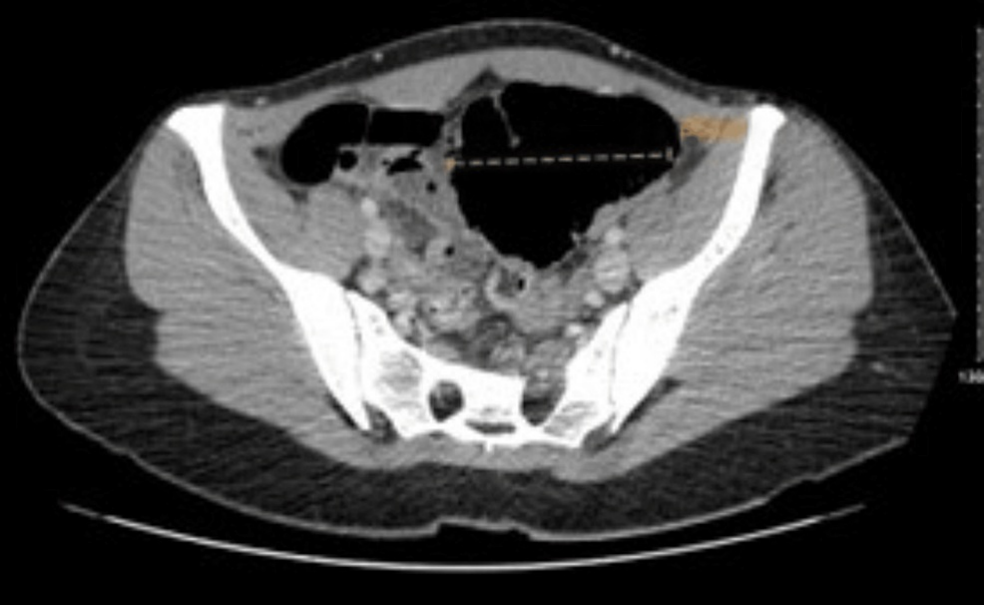
CT scan of abdomen and pelvis with contrast in axial view, showing distended caecum, seen to the left of the midline

**Figure 2:**
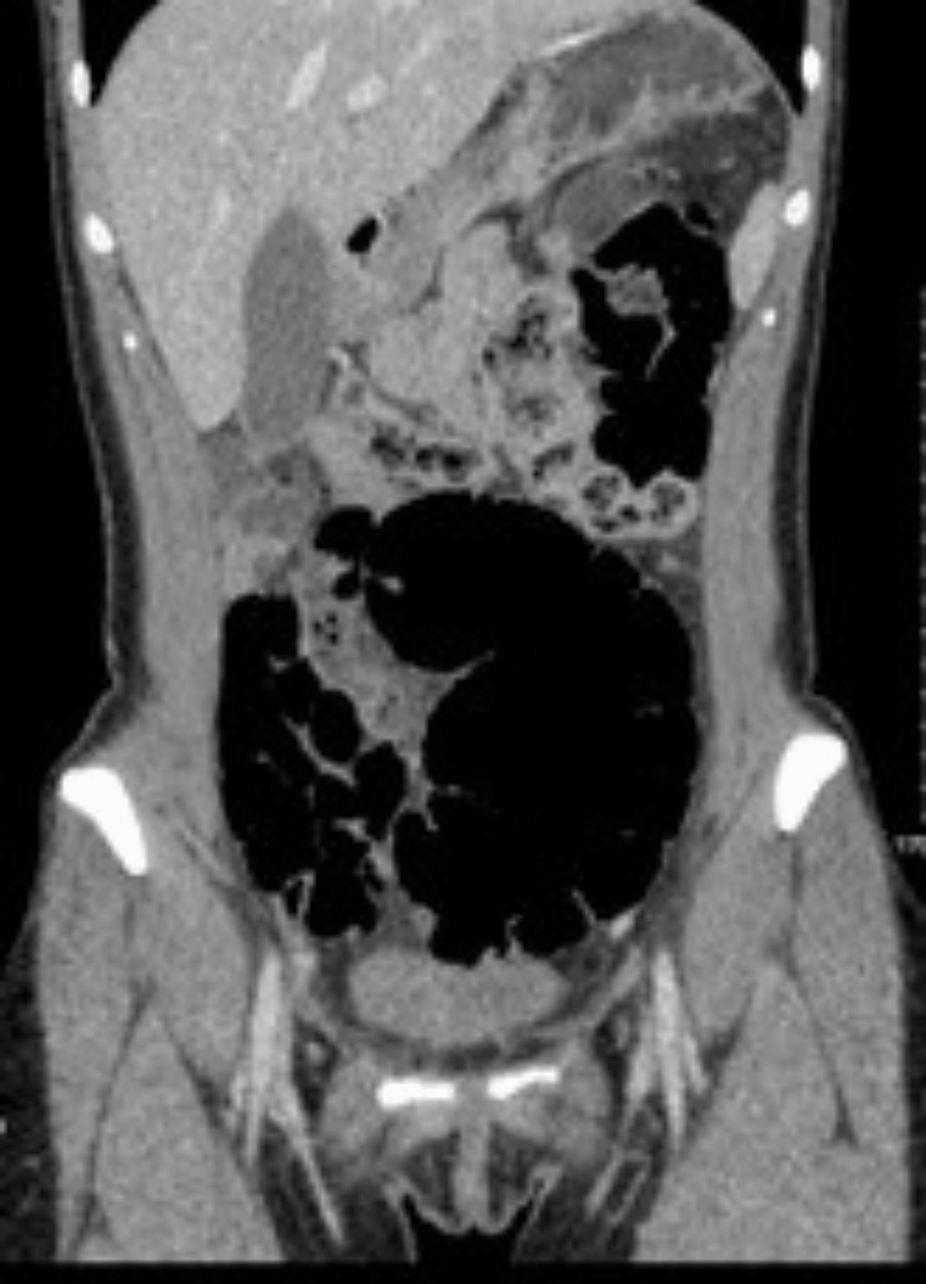
CT scan of abdomen and pelvis in sagittal view showing distended caecum, maximally 8.6cm

We report a case of a 21-year-old female presenting to the Emergency Department with a one-day history of lower abdominal pain, associated with nausea and vomiting. On examination, she was diffusely tender across the lower abdomen, predominantly in the right lower quadrant. Laboratory blood tests revealed elevated white cell count and C-reactive protein (CRP). Of note, this young lady had no significant past medical or surgical history, was not on any medications, and had no family history of bowel pathology. Upon presentation, she further underwent radiological imaging in the form of a plain film of abdomen (PFA) followed by a CT of the abdomen and pelvis, which suggested a distended loop of bowel, most likely caecum, to the left of the midline with volvulus (Figure 1, 2). The caecum was distended maximally at 8.2cm and contained a small amount of faeces; however, there was no fluid level. The small bowel remained undistended in appearance.

Following adequate initial resuscitation with intravenous fluids, administration of analgesia, and antibiotics, she was taken to the operating theatre for surgical management.

**Figure 3:**
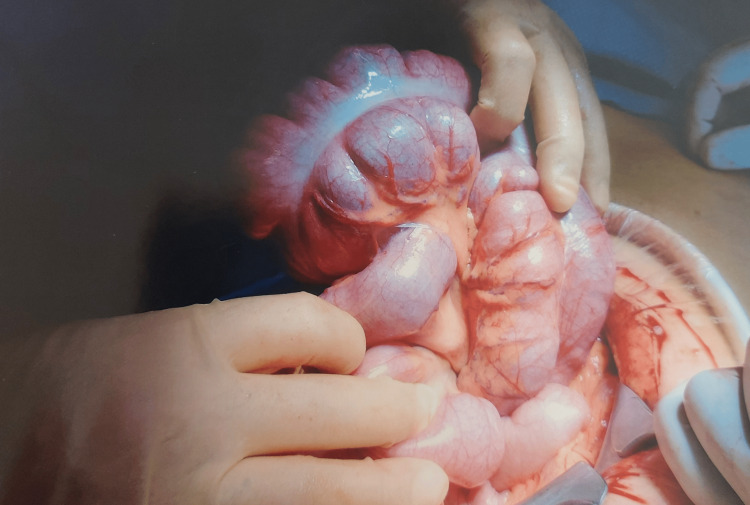
A highly mobile caecum twisted by more than 180 degrees around its axis noted at laparotomy

**Figure 4:**
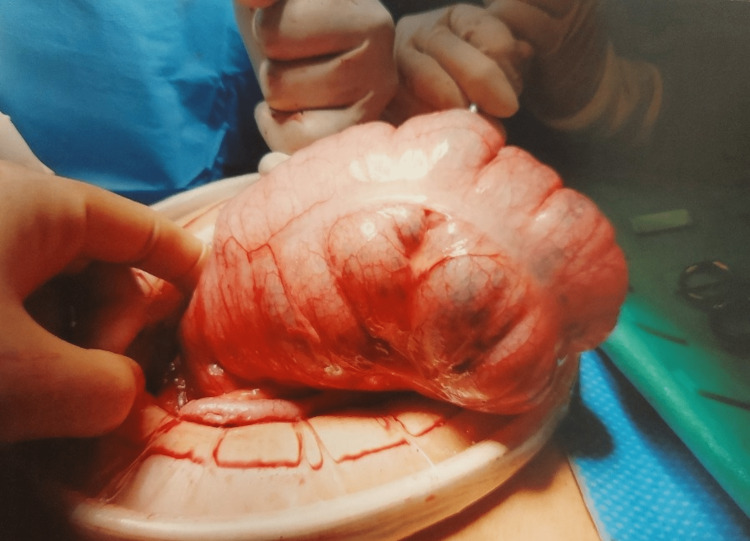
Distended caecum maximally at 8.4cm seen at laparotomy

A midline laparotomy was performed. Intraoperatively, a caecal volvulus was noted, in the form of a highly mobile caecum twisted by more than 180 degrees around its axis (Figure 3). This was grossly distended (Figure 4), however, was viable and the right colon was noted to be free as well. A thorough wash-out was performed. The caecum was untwisted following which an appendicectomy was performed. A lateral wall peritoneal flap was created, and the base of the appendix was fixed to this; furthermore, fixation of ascending colon to lateral wall and caecopexy was performed using 2-0 vicryl suture. The patient made an uneventful post-operative recovery and was discharged five days after surgery. She remained well on follow-up visit in the outpatient clinic, four weeks post-operatively with a complete return to normal activity.

## Discussion

Caecal volvulus is a rare clinical entity that occurs when the segment of bowel gets entangled or encircled upon a mesenteric axis [2]. Caecal volvulus most commonly occurs in female patients in their second or third decade of life [2]. There are three main types of caecal volvulus [2]. Type 1 consists of a clockwise axial twisting or torsion of the caecum along an axis, and type 2 consists of twisting or torsion of a portion of the terminal ileum, leading to an abnormal final site. Together, they make up 80% of caecal volvulus cases. Type 3, also known as a caecal bascule, accounting for 20% of cases, is the cranial folding of the caecum with no axial twisting. Most commonly, the sigmoid colon is affected followed by the caecum [3].

While this pathology is multifactorial, incomplete caecal volvulus development, anatomical variations, malrotation or partial intestinal rotation during developmental embryogenesis are considered to be major contributing factors [4]. Congenital adhesions and Hirschprung's disease are also known etiological factors.

The most well-theoreticized cause of caecal volvulus is related to embryogenesis. This is characterised by a highly mobile caecum and ascending colon being fixed only partially [5].

Surgical intervention is the definitive form of treatment for this condition. The type of operation depends on the duration of onset of symptoms, past medical or surgical history, overall functional status, and appearance of the bowel at laparoscopy or laparotomy. If there is evidence of intestinal gangrene or viability is uncertain, resection is unavoidable.

If the bowel segment is viable, then it may suffice to untwist the affected segment and perform a caecopexy to the lateral wall as done in our case. This could also be done via laparoscopy if an experienced surgeon is present. Alternatively, a caecostomy can be performed. A laparoscopic procedure may be associated with decreased post-operative pain, leading to quicker recovery, and decreased length of stay in the hospital [6]. However, this method is also associated with a higher rate of recurrence [7]. If bowel resection is performed, in the case of caecal volvulus, the operation of choice is a right hemicolectomy and primary ileocolic anastomosis [8]. Whilst being a more definite form of treatment, it may be associated with higher morbidity and increased length of stay in hospital.

## Conclusions

Our case highlights the importance of recognition, resuscitation, diagnosis, and treatment of caecal volvulus in a timely fashion. This is best assessed through an adequate and detailed history and complete clinical examination. Any patient with a high index of clinical suspicion of caecal volvulus must be worked up with routine blood tests and relevant imaging as deemed necessary based on the status and history of the patient. Accordingly, surgical management should be undertaken as discussed above depending on patient pre-operative and intra-operative findings and surgeon experience.

In our opinion, due to the wide range of surgical options for treatment, as discussed previously, an individualised approach should be opted for to ensure that management is tailored according to each individual case. In any case, the patient and their family must be advised of the risk of recurrence and should be counselled about the possible surgical options available. 

This case also highlights the importance of an experienced radiologist in aiding the general surgeon with diagnosis and an eventual satisfactory outcome for the patient.
